# The Effect of *Salvia tomentosa* Miller Extracts, Rich in Rosmarinic, Salvianolic and Lithospermic Acids, on Bacteria Causing Opportunistic Infections

**DOI:** 10.3390/molecules29030590

**Published:** 2024-01-25

**Authors:** Ewelina Piątczak, Joanna Kolniak-Ostek, Weronika Gonciarz, Paweł Lisiecki, Urszula Kalinowska-Lis, Magdalena Szemraj, Magdalena Chmiela, Sylwia Zielińska

**Affiliations:** 1 Department of Pharmaceutical Biotechnology, Faculty of Pharmacy, Medical University of Lodz, Muszyńskiego 1, 90-151 Lodz, Poland; 2 Department of Fruit, Vegetable and Plant Nutraceutical Technology, Wrocław University of Environmental and Life Sciences, Chełmońskiego 37, 51-630 Wroclaw, Poland; joanna.kolniak-ostek@upwr.edu.pl; 3 Department of Immunology and Infectious Biology, Faculty of Biology and Environment Protections, University of Lodz, Banacha 12/16, 90-237 Lodz, Poland; weronika.gonciarz@biol.uni.lodz.pl (W.G.); magdalena.chmiela@biol.uni.lodz.pl (M.C.); 4 Department of Pharmaceutical Microbiology and Microbiological Diagnostics, Faculty of Pharmacy, Medical University of Lodz, Muszyńskiego 1, 90-151 Lodz, Poland; pawel.lisiecki@umed.lodz.pl (P.L.); magdalena.szemraj@umed.lodz.pl (M.S.); 5 Department of Cosmetic Raw Materials Chemistry, Faculty of Pharmacy, Medical University of Lodz, Muszyńskiego 1, 90-151 Lodz, Poland; urszula.kalinowska-lis@umed.lodz.pl; 6 Department of Pharmaceutical Biotechnology, Faculty of Pharmacy, Wroclaw Medical University, Borowska 211, 50-556 Wroclaw, Poland; sylwia.zielinska@umw.edu.pl

**Keywords:** antibacterial activity, cytotoxic activity, MTT, opportunistic infections, polyphenols, rosmarinic acid

## Abstract

Methanolic-aqueous extracts of *Salvia tomentosa* Miller roots, aerial parts, and inflorescences were examined for their content of polyphenolic derivatives and the antimicrobial and cytotoxic effect. In the polyphenolic-rich profile, rosmarinic, salvianolic, and lithospermic acids along with various derivatives were predominant. A total of twenty phenolic compounds were identified using the UPLC/DAD/qTOF-MS technique. These were caffeic acid, rosmarinic acid derivatives, lithospermic acid derivatives, salvianolic acids B, F, and K derivatives, as well as sagerinic acid, although rosmarinic acid (426–525 mg/100 g of dry weight—D.W.) and salvianolic acid B (83–346.5 mg/100 g D.W.) were significantly predominant in the metabolic profile. Strong antibacterial activity of *S*. *tomentosa* extracts was observed against *Staphylococcus epidermidis* (MIC/MBC = 0.625 mg/mL) and *Bacillus cereus* (MIC = 0.312–1.25 mg/mL). The extracts showed low cytotoxicity towards the reference murine fibroblasts L929 and strong cytotoxicity to human AGS gastric adenocarcinoma epithelial cells in the MTT reduction assay. The observed cytotoxic effect in cancer cells was strongest for the roots of 2-year-old plant extracts.

## 1. Introduction

Exposure to environmental pollution, some cleaning agents, and cosmetics has been associated with a significant decrease in immunity among the general population [1]. This can often result in opportunistic infections from generally non-pathogenic microorganisms which do not pose a threat to healthy people and may even play a beneficial role, such as *Staphylococcus epidermidis* or *Bacillus cereus* [2]. Such infections can be particularly severe in people struggling with chronic viral infections such as HIV or fighting cancer.

As such, there is a need to identify substances that can resist infection and maintain the proper condition of the body. Fortunately, many substances of natural origin, such as specialized plant metabolites, appear to be promising candidates. This group comprises an extensive and chemically diverse range of substances that have strong biological effects, both as single compounds and in mixtures with others [3]. These include alkaloids and terpenoids, which show strong biological activity, as well as phenylpropanoids, all of which have been found to possess antimicrobial and anticancer properties when present in plant essential oils and in non-volatile form [4].

The present study examines the phytochemical profile and the antimicrobial and cytotoxic effect of extracts obtained from the roots, aerial parts, and inflorescences of *Salvia tomentosa* Miller (Lamiaceae). The sage genus, *Salvia*, as a whole, is relatively well known, and many of its species are used as pharmaceutical raw materials [5]. *S*. *tomentosa* itself is native to South-East Europe and the Transcaucasus region (Albania, Bulgaria, Greece, Crimea, Lebanon-Syria, and Turkey) [6]. It is a perennial plant that flowers during its second year of vegetation. In Turkish folk medicine, the aerial parts of *S*. *tomentosa* are used as antibacterial and anti-inflammatory agents, as well as against rheumatic, stomach, and abdominal pain [7,8]. Its essential oils are dominated by β-pinene, α-pinene, and camphor [9], as well as 1,8-cineol, β-caryophylene, cyclophenchene [10]. In addition, the extracts from the aerial parts and leaves contain various phenolic acids (e.g., vanillic, galic, rosmarinic, and chlorogenic acids), flavonoids (e.g., rutin, miricetin, quercetin, luteolin, and flavone derivatives), and diterpenoids (7α-acetoxyroyleanone and 7α-hydroxyroyleanone, royleanone, and 6,7-dehydroxyroyleanone) [7,11,12]. The main compounds are known to possess antioxidant, anti-inflammatory, antibacterial, and antiviral properties and are promising anticancer drugs against various human cancer types [13,14,15].

Previous studies have only examined the essential oils of the plant and extracts of its aerial parts using antimicrobial and antioxidant assays [9,10], and no detailed study has been performed on the roots or inflorescences. However, several di- and triterpenoids have been noted in acetone extracts from roots [16]. To address this gap, the present study performs a detailed investigation of the profile of metabolites present in methanolic-aqueous extracts of *S*. *tomentosa* roots, aerial parts, and inflorescences using UPLC/DAD/qTOF-MS. The study also investigates the antimicrobial properties of the extracts and tests their cytotoxic properties.

## 2. Results and Discussion

### 2.1. Phytochemical Studies

#### 2.1.1. Qualitative Analysis

The roots and aerial parts of the one- and two-year-old plants (R1, R2, and AP1 and AP2, respectively) and the inflorescences of the two-year-old plants (I) were examined for specialized metabolites. 

In total, 20 phenolic compounds were identified in the *S*. *tomentosa* extracts (Table 1 and Table 2). These included rosmarinic acid (RA), rosmarinic acid derivatives (RAd), salvianolic acid derivatives (SAd), and lithospermic acid derivatives (LAd), as well as cyclobutene lignan–sagerinic acid and free caffeic acid (CA). 

**Figure 1:**
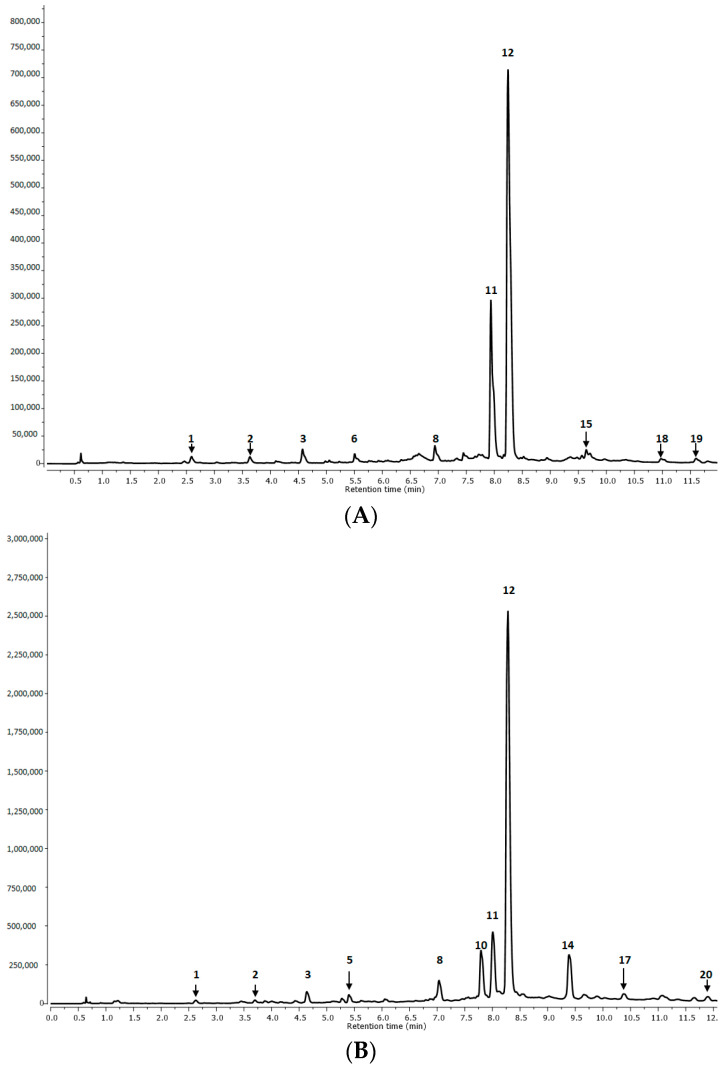

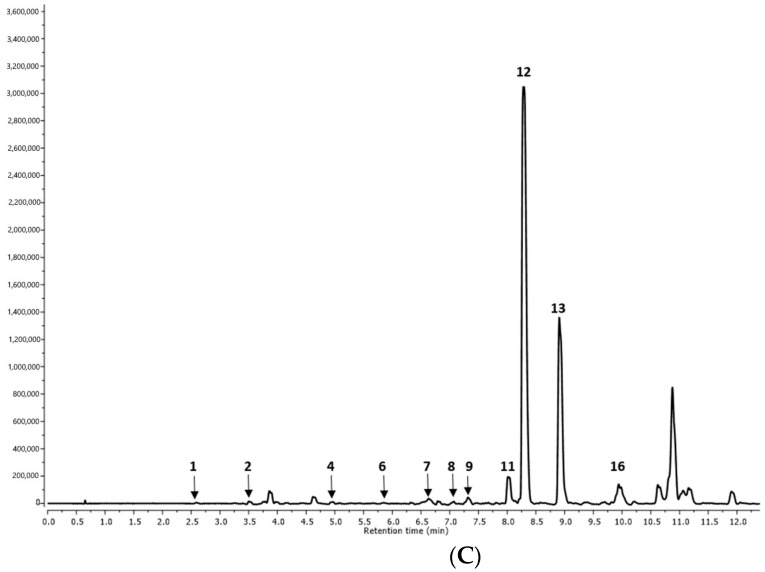
UPLC-DAD chromatogram at 280 nm of *S*. *tomentosa* methanolic-aqueous extracts from roots (**A**), aerial parts (**B**), and inflorescences (**C**). Peak number identities are displayed in Table 1.

**Figure 2:**
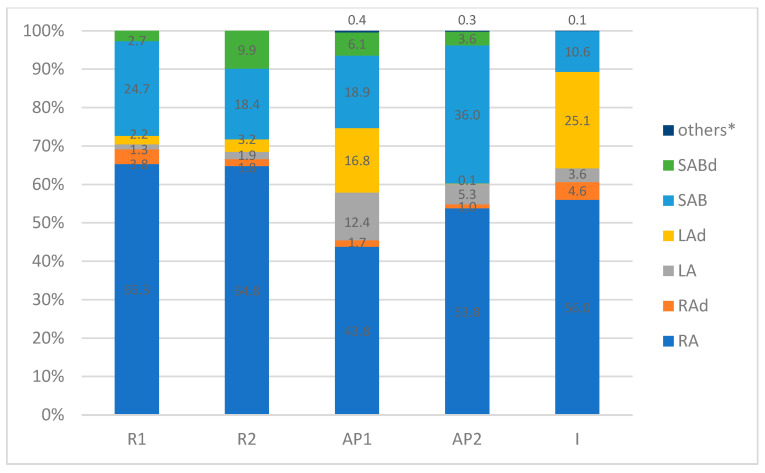
The content (%) of the main components in the various organs of *S*. *tomentosa* plants. * The sum of the percentages of ingredients whose individual content in the extract is less than 0.4%; R1—roots of 1-year-old plants; R2—roots of 2-year-old plants; I—inflorescences from 2-year-old plants; AP1—aerial parts from 1-year-old plants; AP2—aerial parts from 2-year-old plants. SABd—salvianolic acid B derivatives; SAB—salvianolic acid B; LAd—lithospermic acid derivatives; LA—lithospermic acid; RAd—rosmarinic acid derivatives; RA—rosmarinic acid.

The RA (peak no **12**), i.e., the salvianic ester of caffeic acid, exhibited a deprotonated molecular ion at *m*/*z* 359, whose fragmentation led to characteristic ions at 197, 179, 161, and 135 (*m*/*z*). These peaks correspond to a deprotonated form of salvianic acid (danshensu or 3-(3,4-dihydroxyphenyl)lactic acid), a deprotonated CA, a dehydrated form of the mentioned acids, and a residue of CA after decarboxylation (Figure 1 and Figure 2; Table 1 and Table 2). These results were in agreement with the fragmentation described by Barros et al. [17], Katanić et al. [18], and Lecomte et al. [19]. Similarly, RA has also been found to be the predominant compound in numerous other *Salvia* species [16,17,18,19,20]. 

The RA derivatives (RAd) included three hexosides of RA with the same molecular ions [M-H]^−^ at *m*/*z* 521 (peaks no **2**, **3,** and **9**). Their corresponding fragmentation ions at *m*/*z* 359 and (197, 179, 161, and 135) derived from rosmarinic acid (359) (the main component of its fragmentation), resulting from a loss of hexoside moiety (−162 u) from a molecular ion at *m*/*z* 521. Rosmarinic acid hexosides have been previously identified as the predominant ingredients in *S*. *viridis* [20], *S*. *bulleyana* [21], *S*. *officinalis* [22], *S*. *blepharochlaena* [23], *S*. *viridis* [24], and *S*. *cadmica* [25].

The second most abundant group of compounds present in the extracts was isomers and derivatives of salvianolic acids. These included salvianolic acid A (peak no **16**), salvianolic acid B (SAB) (peaks no **11**, **17,** and **18**), salvianolic acid F (peaks no **19** and **20**) and salvianolic acid K (peaks no **4**, **6**, **7,** and **15**) (Table 1 and Table 2, Figure 1 and Figure 2). 

Compound **16** (Table 1 and Table 2) found in the inflorescences was identified as salvianolic acid A (SAA) with a characteristic parent ion at *m*/*z* 493. Its fragmentation yielded a product ion typical for RA (359) and loss of fragments at *m*/*z* 197 and 179. SAA has also been found in the root extracts of *S*. miltiorrhiza [26,27]. 

Peak **11** (Rt = 11.08 min), identified as SAB, was present in all tested plant organs. It presented its main ion at *m*/*z* 717 and the following fragmentation ions at *m*/*z* 537, 519, 339, 321, 295, 197, and 179. The typical fragmentation ion for SAB after the cleavage of two danshensu units was the ion at *m*/*z* 321. The removal of salvianolic and caffeic acids from the SAB of mass 718 Da, [M-H-198-180]^−^, resulted in the formation of a fragmentation ion of *m*/*z* 339, the further decarboxylation of which (−44 u) may lead to ion formation at *m*/*z* 295. 

Compound **17** (Rt = 10.37 min), present in the aerial parts, was identified as one of the two SAB isomers. The fragmentation ion at *m*/*z* 537 can be attributed to the loss of a caffeic acid (−180 u) unit from the parent ion at *m*/*z* 717. The remaining peaks at *m*/*z* 359 and 179 were analogous to those present in compound **11**, which was identified as SAB. 

Another isomer of SAB identified in the extracts in the present study was compound **18,** which was only detected in the extracts of *S*. *tomentosa* roots. Its main fragmentation ion at *m*/*z* 519 can be attributed to the loss of a danshensu unit (−198 u) from the parent ion at *m*/*z* = 717. The compound showed *m*/*z* 359, with the characteristic loss of a fragment corresponding to the caffeic acid residue (−162 u). SAB isomers were one of the most abundant compounds in other *Salvia* species, for example *Salvia miltiorrhiza* [28]. SAB isomers have also previously been found in *S*. *africana*, *S*. *officinalis ‘Icterina’,* and *S*. *mexicana* aqueous extracts [29], and in *S*. *apiana* and *S*. *farinacea* var. *Victoria Blue* decoctions [30]; however, they were present in smaller amounts compared to the *S*. *tomentosa* methanolic-aqueous extracts described in our study. 

In turn, compounds no **4** and **6** (Table 1 and Table 2, Figure 1), detected in the inflorescences and roots, showed parent ions at *m*/*z* 555 and identical fragmentation ions at 537, 359, 295, and 179. They were identified as hydrated salvianolic acids K (SAK) due to the fragmentation pattern with successive losses of danshensu (−197 u) and caffeic acid (−179 u or −161 u) moieties. Another SAK derivative (peak no 7) was found at Rt = 6.50 min, and its parent ion *m*/*z* 683 corresponded to salvianolic acid K methyl pentoside. The loss of the methyl-pentoside residue (−146 u) from the main molecular ion showed the presence of a fragmentation ion typical for SAK at *m*/*z* 537. All SAK derivatives were absent in the aerial parts extracts of *S*. *tomentosa*.

Peaks **19** and **20** (Rt = 11.79 and 11.89 min, respectively) represented by the same molecular ion ([M-H]^−^ at *m*/*z* 313) were assigned to salvianolic acid F (SAF) isomers. Compound **20**, found in the aerial parts, has only one fragment ion at *m*/*z* 161, suggesting the decomposition of the main molecule to the dehydrated form of caffeic acid (CA). The second compound, **19**, found only in inflorescences, possesses more fragmentation ions, i.e., at *m*/*z* 269, 203, 161, and 135: these may represent a decarboxylated form of SAF (269 u), an SAF residue after the loss of 3,4-dihydroxyphenyl (203 u), a dehydrated form of CA (161 u), and a decarboxylated form of CA (135 u). SAF isomers have been detected in the roots of *S*. *viridis* [20], *S*. *cadmica* [25], and *S*. *bulleyana* [27], and in the aerial parts of *S*. *verticillata* subsp. *amasiaca* [23], *S*. *blepharochlaena* [23], and *S*. *cadmica* [25].

Four compounds, represented by peaks **8** and **13**–**15** were annotated as lithospermic acid derivatives (LAd) (Table 1 and Table 2, Figure 1). Peak no **8** was present in all analyzed extracts and was attributed to lithospermic acid (LA). Compound **15** (Rt = 9.72), detected in the roots, was annotated as an LAd with parent ion *m*/*z* 569. Peaks **13** and **14** (Rt = 8.91 and 9.38 min, respectively) that showed parent ion *m*/*z* 1074, were identified as dimers of lithospermic acid and were detected in aerial parts only. The MS/MS fragmentations yielded product ions at *m*/*z* 537 due to the loss of lithospermic acid A (537 Da) and fragments with *m*/*z* 359, 197, and 179. Some isomeric lithospermic acids were identified previously in *S*. *miltiorrhiza*, *S*. *cadmica*, *S*. *cavaleriei*, *S*. *chinensis*, and *S*. *sonchifolia* but were not present as dimers in any of these plants [22,25,31]. 

Moreover, free caffeic acid (CA, peak no **1**) [*m*/*z* 179] was also found in all plant parts (Table 1, Figure 1). This compound could be the product of the hydrolysis of more complex structures like rosmarinic, lithospermic, or salvianolic acids. CA has been previously identified in the hydromethanolic extract of the roots and aerial parts of *S*. *cadmica* [25] and in South African *Salvia* species such as *Salvia africana-lutea*, *S*. lanceolata, and *S*. chamelaeagnea; these are used to treat fever and some inflammation [32].

Apart from phenylpropanoid derivatives, a cyclobutene lignan–sagerinic acid (peak no **10**) (Rt = 7.80 min) with a parent ion at *m*/*z* 719 and a characteristic loss of fragments [*m*/*z* 359, 197, 179, 161, 135] was also identified in the aerial parts of *S*. *tomentosa*. These annotations were previously found in *Rosmarinus officinalis*, and three red-colored basil cultivars [33,34].

#### 2.1.2. Quantitative Analysis

The methanolic-aqueous extracts of *S*. *tomentosa* roots, aerial parts, and inflorescences were found to be rich sources of phenylpropanoid compounds (Table 2). The phytochemical profile of all extracts was significantly predominated by RA (425.87–524.71 mg/100 g D.W.). A number of RA derivatives were also found, which, together with RA, accounted for up to 69% of the total share of all compounds (Figure 2). However, only 1.0–4.6% of RA was present in the glycoside and hydrated forms (Table 2, Figure 2).

Moreover, all extracts were relatively rich in SAB, its derivatives (SAd), and lithospermic acid (LA) dimers. SAB together with SAd accounted for 10.6% to 39.5% of the total composition, with the highest contents found in the aerial parts and roots (24.9–39.5% and 27.4–28.3%, respectively; Figure 2). In turn, LA and its derivatives were found in considerable amounts (28.7–29.2%) in the aerial parts, including inflorescences (Table 2, Figure 2). The presence of the remaining compounds was negligible, which was less than 0.4% (Table 2, Figure 2).

### 2.2. Antimicrobial Studies

The antimicrobial activity of the methanolic-aqueous extracts from *S*. *tomentosa* was tested against 14 Gram-positive bacterial strains, six Gram-negative bacterial strains, and two fungal strains. The extracts from both the aerial parts and roots demonstrated moderate inhibitory and biocidal effects against most of the tested strains compared to the routinely used antibiotics and fungicides (Table 3), demonstrating strong antibacterial effects against *Staphylococcus epidermidis* and *Bacillus cereus* (Table 3). Minimal inhibition concentration (MIC) and minimal bactericidal concentration (MBC) were obtained against *Staphylococcus epidermidis* when 0.625 mg/mL of the root, aerial part, and inflorescence extracts was used. In the case of *Bacillus cereus*, more potent effects were obtained from the root and inflorescence extracts (MIC/MBC = 0.312 mg/mL) than from the non-flowering aerial parts (MIC/MBC = 1.25 mg/mL). Previous studies on methanolic extracts of the aerial parts did not indicate any antimicrobial activity against *Bacillus cereus* [9]. Other studies have examined the antibacterial potential of essential oils from the non-aerial parts [9,10,35]; however, the results cannot be compared with ours due to methodological differences. 

Both *S*. *epidermidis* and *B*. *cereus* are Gram-positive bacteria. Our present findings confirm those of other authors [25,36,37], indicating that extracts or essential oils from other plant species, including genus *Salvia*, are more active against Gram-positive than Gram-negative bacteria. This could be related to the cells lacking an external membrane, which may act as a barrier against antibacterial macromolecules [38]. Alternatively, Gram-positive strains may demonstrate greater ion permeability and leakage of intracellular components from bacterial cells or impaired bacterial enzyme systems [39]. 

*B*. *cereus* and *S*. *epidermidis* are opportunistic pathogens, indicating that they may induce severe infections in immunocompromised patients. *B*. *cereus* is a spore-forming bacterium, which allows it to survive longer in extremes of temperature. These bacteria are commonly found in the environment and can contaminate food such as beef, turkey, rice, beans, and vegetables. *B*. *cereus* can multiply rapidly at room temperature and produce toxins that can cause food poisoning, resulting in diarrhea and vomiting (emesis). The diarrheal illness is often related to meat, milk, vegetables, and fish; the emetic illness, in contrast, is typically associated with rice products but has also been related to other products such as potatoes, pasta, and cheese. Infections caused by *B*. *cereus* are usually self-limiting and do not require specific treatment, but in immunocompromised patients, antibiotic therapy is often necessary. *B*. *cereus* is also associated with infections of the eyes, respiratory tract, and wounds [40,41,42]. Most importantly, *B*. *cereus* is resistant to two major classes of antibiotics: trimethoprim/sulfamethoxazole and beta-lactam antibiotics [43]. Therefore, natural extracts exhibiting antibacterial activity may be useful in therapy.

*Staphylococcus epidermidis* forms the main part of the microbiota of the skin and mucous membranes. It is one of the most often isolated bacteria in hospitals and is commonly responsible for nosocomial infections of the bloodstream, cardiovascular system, eye, ear, nose, and throat. Its pathogenicity is mainly due to its ability to form biofilms [44,45]. A biofilm is a complex, three-dimensional structure consisting of a community of microorganisms surrounded by an exopolymer substance called a matrix. The exopolymer makes up 50–90% of the total organic mass of the biofilm and consists of extracellular DNA (eDNA), proteins, lipids, polysaccharides, divalent cations, and water channels. The matrix serves as a protective barrier against external factors, acts as a source of nutrients and enzymes, and enables the transport of intercellular substances [46]. As *S*. *epidermidis* is protected against attacks from the immune system and antibiotic treatments when in the biofilm, and such infections are difficult to eradicate. Approximately 80% of *S*. *epidermidis* strains from nosocomial infections are resistant to methicillin, and most are also resistant to other antibiotics [44,45]. Therefore, *S*. *tomentosa* extracts, demonstrating antibiotic potential, may be of value in countering such infections, especially in immunocompromised patients. 

### 2.3. Cytotoxicity Studies

**Figure 3:**
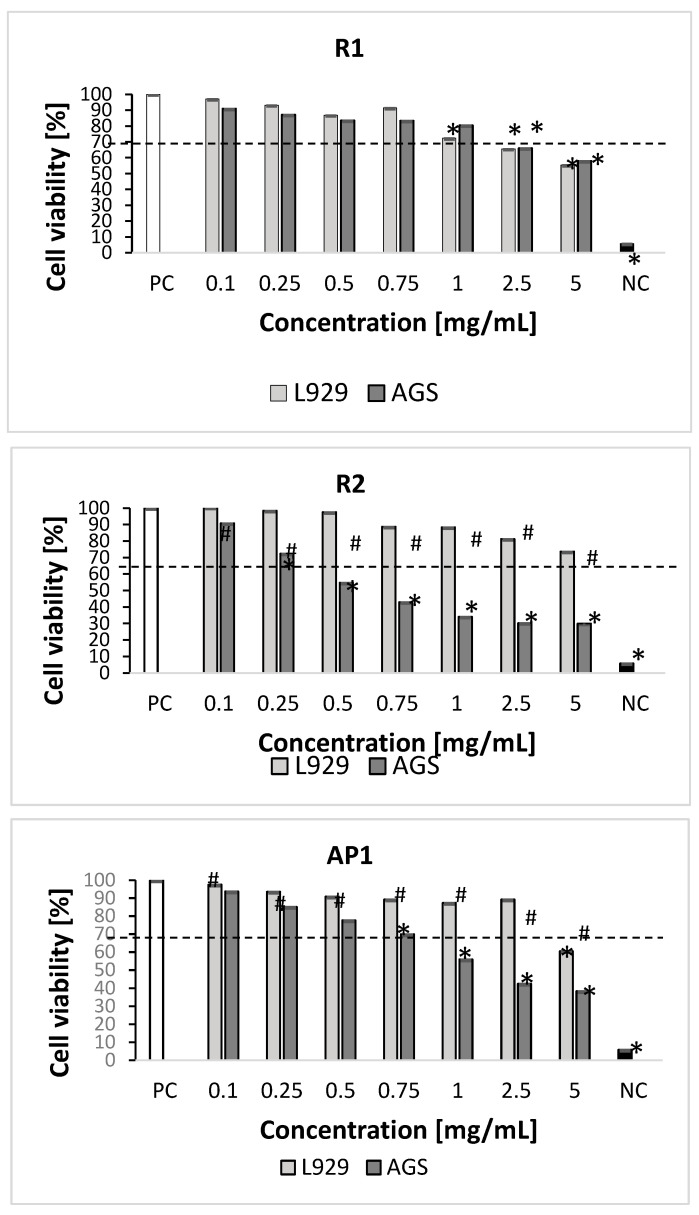

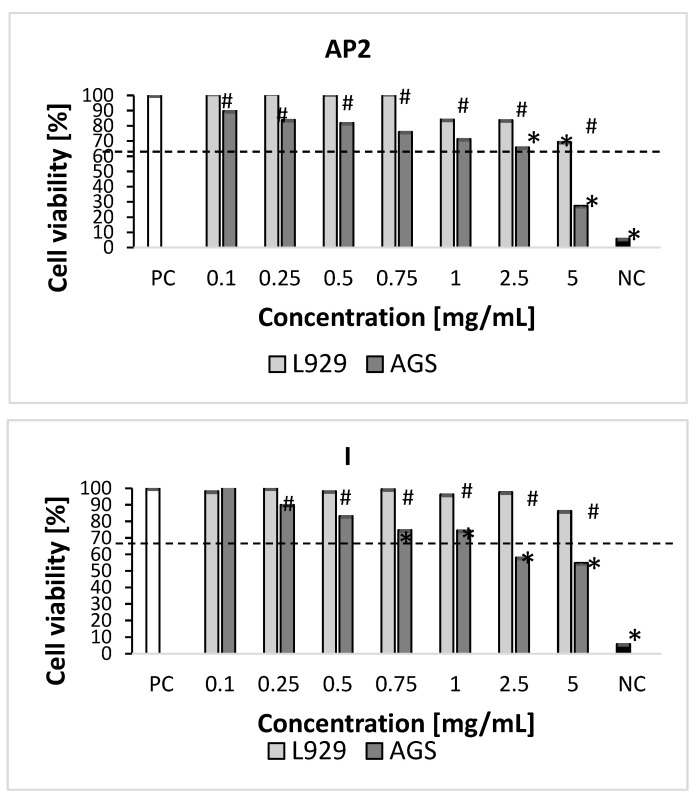
Cytotoxic effect of *S*. *tomentosa* plant extracts towards L929 and AGS cells. The cytotoxicity was assessed by MTT [(3-(4,5-dimethylthiazol-2-yl)-2,5-diphenyltetrazolium bromide)] reduction assay. Complete RMPI-1640 medium (cRPMI) was used as a positive control (PC) of cell viability (100% viable cells) and 0.03% H_2_O_2_ as a negative control (NC) of cell viability (100% dead cells). Statistical significance: *p* < 0.05; * untreated cells vs. cells treated with tested plant extracts; # L929 cell line vs. AGS cell line (Mann–Whitney U-test). R1—roots of 1-year-old plants; R2—roots of 2-year-old plants; AP1—aerial parts from 1-year-old plants; AP2—aerial parts from 2-year-old plants; I—inflorescences from 2-year-old plants.

The methanolic-aqueous extracts from the aerial parts (one-year and two-year-old plants) in the highest concentration (5 mg/mL) significantly diminished the growth of L929 fibroblasts (30–39% of dead cells); however, the inflorescence extract did not show any cytotoxic activity at any of the tested concentrations (Figure 3). The root extract of one-year-old plants at the highest concentrations (5 mg/mL, 2.5 mg/mL, and 1 mg/mL) had a stronger inhibitory effect on L929 cell metabolic activity (28–45% of dead cells) than those of the two-year-old plants; the latter only reduced cell viability at the highest concentration (5 mg/mL) (27% of dead cells). 

The root extract from the two-year-old plants also demonstrated the strongest inhibitory effect on the metabolic activity of AGS cells at the concentration range of 0.25–5 mg/mL (28–70% of dead cells). The root extract from the one-year-old plant significantly inhibited AGS cell growth at the two highest concentrations (2.5 and 5 mg/mL). 

The extracts from inflorescences and aerial parts exhibited significantly greater cytotoxic activity against AGS cells in the range of 0.75–5 mg/mL (25–46% and 30–62% of dead cells, respectively) (Figure 3). 

None of the inflorescence extracts demonstrated toxicity towards the reference mouse L929 fibroblasts at any tested concentrations. Similarly, no such cytotoxicity was noted for extracts from aerial parts and roots (two-year-old plants) at 2.5 mg/mL and below, or for root extracts (one-year-old) below 1 mg/mL. 

Kirmizibekmez et al. [47] found various *Salvia* extracts, including *S*. *tomentosa*, to demonstrate low or absent cytotoxicity towards L6 mammalian cells. Similarly, hydromethanolic aerial and root extracts from *S*. *cadmica* at concentrations of 2.5 mg/mL and below did not demonstrate any cytotoxicity against the same cell line (L929) based on MTT assay [25]. Abu-Dahab et al. [48] also report that the extracts of the *Salvia* species *S*. *fruticosa*, *S*. *horminum,* and *S*. *syriaca* did not appear cytotoxic towards various normal cell lines in the concentration range 0.1 and 100 μg/mL. 

In the present study, non-neoplastic cells like L-929 fibroblasts were found to be resistant to studied extracts. Only R1 and AP1 extracts diminished cell viability below 70%, at concentration ranges of 5–1 mg/mL and 5 mg/mL, respectively. Hence, the developed extracts appear to be very safe in contact with normal cells, which bodes well for their use in the development of formulations with antimicrobial or pro-regenerative effects. However, the root extracts (two-year cultivation; concentration: 0.25–5 mg/mL), inflorescence, and leaf extracts (concentration: 0.75–5 mg/mL) were found to be cytotoxic against the studied gastric cancer AGS cells. Therefore, *S*. *tomentosa* extracts may be of value in anticancer treatment. However, further research is needed in this area to explore their mechanism of action. Indeed, Berk et al. [49] report *S*. *tomentosa* extracts to have cytotoxic effects on hepatocellular carcinoma HepG2 cells (IC_50_ = 0.417 mg/mL). 

Other *Salvia* extracts have also exhibited cytotoxic activity against different cancer cell lines. For example, Firuzi et al. [50] found that *S*. *eremophila* methanolic and hydromethanolic extracts significantly diminished the viability of breast cancer MCF-7 cell lines and increased the effectiveness of the reference drug cisplatin. Jiang et al. [51] report that ethanolic and acetone extracts from *S*. *miltiorrhiza* roots displayed high cytotoxic potential against HepG2 cells (IC_50_ = 17.3 and 83.2 µg/mL, respectively), as did extracts of *S*. *officinalis* roots and leaves (IC_50_ = 19.6–43.8 µg/mL and 64.4–90.0 µg/mL, respectively). Uysal et al. [52] showed that the methanolic and aqueous extracts of *S*. *ceratophylla* roots caused the death of HepG2 cells (30.9 and 34.5% of cell viability), while aqueous extracts from the aerial parts were more active against B16 4A5 cells (57.3% of cell viability). 

Our present findings suggest that the cytotoxic activity of the tested plant extracts against AGS cells may be related to their phytochemical profile, with high amounts of polyphenols. Various studies based on animal models have found plant polyphenols to inhibit tumorigenesis and arrest cancer cell proliferation, potentially via the modulation of numerous signaling pathways dependent on nuclear factor B (NF-kB) or mitogen-activated protein kinase (MAPK). These pathways are related to cell autophagy, apoptosis, and inflammation [53,54,55]. Parsaee et al. [56] found *S*. *chorassanica* root extracts to have pro-apoptotic activity against human HeLa cells, which suggests that plant polyphenols can influence the course of an apoptotic process [57]; however, this thesis has yet to be confirmed. 

## 3. Materials and Methods 

### 3.1. Plant Material

*S*. *tomentosa* seeds were obtained from the Botanical Garden of Medicinal Plants, Wroclaw Medical University (Wroclaw, Poland) in 2018. They were sewn in the experimental plot at the Botanical Garden of Medicinal Plants, Medical University of Lodz (Lodz, Poland) (51°77′ N, 19°49′ E). The resulting plants were cultivated for two years and harvested during each vegetation season. A voucher specimen (no EP-ST-2019) was deposited at the Department of Pharmaceutical Biotechnology, Medical University of Lodz (Lodz, Poland). The plant material was collected and dried and then separated into aerial parts and roots. As the plant blooms only in the second year of vegetation, the collected inflorescences were analyzed separately. 

### 3.2. Extract Preparation

Dried and powdered plant material (100 mg for phytochemical studies and 500 mg for antimicrobial and cytotoxic activities, separately) was pre-extracted with chloroform (20 or 100 mL) for 12 h at room temperature. Chloroform supernatants were filtered off. The defatted dried plant material was extracted three times with methanol:water solution at a ratio of 8:2 (*v*/*v*) according to the method described by Piątczak et al. [25]. The obtained dry extracts were stored at 4 °C until used.

### 3.3. Phytochemical Studies

Phytochemical analyses were performed using UPLC equipped with quadrupole Time of Flight—double mass spectrometry detector (UPLC/DAD/qTOF-MS/MS) (Waters Corporation, Milford, MA, USA) as described by Zielińska et al. [58]. Polyphenolic compounds were separated by a UPLC BEH C18 column (1.7 μm, 2.1 × 100 mm, Waters Corporation, Milford, MA, USA). The mobile phase consisted of solvent A (0.1% formic acid in LC–MS grade water, *v*/*v*, Merck, Darmstadt, Germany) and solvent B (0.1% formic acid in LC–MS grade acetonitrile, Merck Germany). 

The retention times and spectra of the analyzed compounds were compared with those of authentic standards. Caffeic, rosmarinic, salvianolic acid B, salvianolic acid F, and its derivatives were quantified with their own standards. Salvianolic acid K and lithospermic acid derivatives were quantified as salvianolic acid A equivalents. The quantities of compounds were expressed as mg per 100 g of plant material dry weight (mg/100 g DW).

### 3.4. Antimicrobial Studies

The antimicrobial activity of the prepared extracts was investigated against fourteen strains of Gram-positive bacteria: *Staphylococcus aureus* ATCC 25923, *S*. *aureus* MM3 MRSA, *S*. *aureus* ZMF MLS_B_, *Staphylococcus hominis* DSM 20328, *Staphylococcus epidermidis* ATCC 12228, *Staphylococcus haemolyticus* PCM 2113, *Staphylococcus simulans* DSM 20723, *Staphylococcus pseudintermedius* PCM 2791, *Enterococcus faecium* PCM 1859, *Enterococcus hirae* ATCC 10541, *Enterococcus faecalis* ATCC 29212, *Bacillus subtilis* ATCC 6635, *Bacillus cereus* PCM 1948, and *Listeria monocytogenes* PCM 2191; six Gram-negative strains: *Escherichia coli* ATCC 25922, *E*. *coli* ZMF ESBL, *Proteus vulgaris* CCM 1799, *Salmonella enteritidis* ZMF 279, *Shigella sonnei* PCM 2466 FII, and *Pseudomonas aeruginosa* ATCC 27833; and two fungal strains: *Candida albicans* ATCC 10231 and *Aspergillus brasiliensis* ATCC 16404. 

The *S*. *haemolyticus* PCM 2113, *S*. *pseudintermedius* PCM 2791, *E*. *faecium* PCM 1859, *B*. *cereus* PCM 1948, *L*. *monocytogenes* PCM 2191, and *S*. *sonnei* PCM 2466 FII strains were taken from the Polish Collection of Microorganisms (PCM). The *P*. *vulgaris* CCM 1799 strain was obtained from the Czech Collection of Microorganisms. The *S*. *hominis* DSMZ 20328 and *S*. *simulans* DSMZ 20723 were obtained from the German Collection of Microorganisms and Cell Cultures (DSMZ). Four bacterial strains, *S*. *aureus* MM3 MRSA, *S*. *aureus* MLS_b,_ *E*. *coli* ZMF ESBL, and *S*. *enteritidis* ZMF 279, were derived from the collection of the Department of Pharmaceutical Microbiology and Diagnostic Microbiology of the Medical University of Lodz. The rest of the strains were received from the American Type Culture Collection (ATCC), Rockville, MD, USA. 

All tested microorganisms were stored at −80 °C in 15% glycerol stocks. Before the experiment, the bacterial strains were transferred to the Mueller–Hinton agar medium (Oxoid, Thermo Fisher Scientific, Waltham, MA, USA) and cultured overnight at 37 °C. Fungal strains were transferred on agar RPMI-1640 medium and cultured for two days at 30 °C. 

The antimicrobial activity of the methanolic-aqueous extracts from the aerial parts, roots, and inflorescences of *S*. *tomentosa* were determined based on MIC (minimum inhibitory concentration) and MBC/MFC (minimum bactericidal concentration/minimum fungicidal concentration) using the broth microdilution method. These assays were performed in the Mueller–Hinton liquid media for bacteria or RPMI-1640 for fungi. All tests were performed in 96-well microtiter plates (Kartell Labware, Noviglio, Italy) according to the European Committee on Antimicrobial Susceptibility recommendations (EUCAST) as described by Grzegorczyk-Karolak et al. [59]. The extracts were used to create a two-fold dilution series in a growth medium in the range of 5 mg–0.009 mg/mL. Gentamicin, fluconazole, and rosmarinic acid were used as positive controls.

### 3.5. Cytotoxicity Studies

#### 3.5.1. In Vitro Cell Cultures

The in vitro cytotoxicity assays were performed using mouse fibroblast L929 cell line (LGC Standards, Middlesex, UK), according to ISO 10993-5 [60], and human gastric adenocarcinoma epithelial cells (AGS) (CRL-1739, ATCC, Rockville, MD, USA). The cells were maintained under standard conditions in complete culture medium (cRPMI 1640 medium) supplemented with fetal bovine serum (FBS) (10%), L-glutamine (2 mM, 1%), and penicillin (50 U mL^−1^)/streptomycin (50 μg mL^−1^) (1%) (Sigma-Aldrich, Darmstadt, Germany). The cultures were incubated in a humidified atmosphere at 37 °C and 5% CO_2_ and cultured according to Kamizela et al. [61]. Cell viability was assessed by exclusion of trypan blue, 93 to 95%.

#### 3.5.2. Measurement of Cellular Metabolic Activity and Cell Growth Inhibition

The metabolic activity of both cell lines was tested in cell cultures in vitro after the application of *S*. *tomentosa* extracts. Cells suspended in complete culture medium (cRPMI-1640) were seeded (2 × 10^5^ cells/well) in 96-well plates for 24 h at 37 °C and 5% CO_2_. The tested extracts were diluted in cRPMI-1640 medium in concentrations of 5, 2.5, 1, 0.75, 0.5, 0.25, and 0.1 mg/mL, and added to the wells with the cells (100 μL/well). Cells were incubated under standard conditions for 24 h. Following incubation, cell morphology was determined via light microscopy, as recommended by ISO norm 10993-5 [60]. Cell metabolism was estimated according to [56] using MTT assay, as recommended by the Food and Drug Administration (FDA) and the International Organization for Standardization (IOS). 

### 3.6. Statistical Analysis

The phytochemical analyses were performed in triplicate. All data are presented as mean values ± standard error (SE). Antibacterial and antifungal tests were repeated twice. Cell viability was determined using an MTT reduction assay (cytotoxicity studies) in four experiments. Statistical significance was considered at a *p* value of <0.05 using the Wilcoxon signed rank test (cytotoxicity assays) and Tukey’s HSD test (phytochemical analysis). Statistical analyses were performed using STATISTICA 13.3 PL software (TIBCO, Wroclaw, Poland).

## 4. Conclusions

Methanolic-aqueous extracts from different organs of field-grown *S*. *tomentosa* appear to be rich sources of rosmarinic acid, salvianolic acids, and lithospermic acid A derivatives. The metabolite profile was not influenced by the developmental stage of the plants harvested from two consecutive vegetation seasons. No significant differences in the metabolite profile of the roots and aerial extracts were noted between harvest periods. However, it is important to note that the inflorescences, which naturally appear in the second year of vegetation, contained considerable amounts of salvianolic acid B and K derivatives, as well as rosmarinic acid glycosides. 

The studied extracts demonstrated strong antimicrobial activity against opportunistic bacterial species, such as *Staphylococcus epidermidis* and *Bacillus cereus*. This result is particularly important in the face of increasing antibiotic resistance and the need to identify alternative treatments for infections. Cytotoxic investigations on mouse fibroblasts indicated that all of the tested extracts were non-toxic against mammalian cells up to a concentration of 5 mg/mL, depending on the plant organ, but showed cytotoxicity against gastric cancer cells (AGS) across a broad concentration range. The extract of roots collected from the second year of cultivation showed the highest cytotoxic activity. 

Our findings significantly improve the knowledge of the metabolite content of *S*. *tomentosa* and their acquisition for further use. As such, *S*. *tomentosa* merits further consideration as a potential herbal medicine suitable for the treatment of infections and for the support of anticancer therapy. Further detailed investigations are recommended into the isolation and study of these compounds in an intact and sustainable form, which is needed for their strong bioactivity. 

## Figures and Tables

**Table 1:** UPLC-DAD ESI-MS detection and identification of phenolic compounds in methanolic-aqueous extracts from *Salvia tomentosa* different organs.

Peak No.	RT (min)	MS [M-H]^−^	MS/MS/Fragmentation Ions [M-H]^−^	Tentative Identification	R1	R2	AP1	AP2	I
1.	2.62	179		Caffeic acid	p	p	p	p	p
2.	3.69	521.0880	359, 197, 179, 161, 135	Rosmarinic acid hexoside I	p	p	p	p	n.d.
3.	4.63	521.1452	359, 179, 135	Rosmarinic acid hexoside II	p	p	p	p	n.d.
4.	4.95	555.1432	537, 359, 295, 179	Salvianolic acid K derivative	n.d.	n.d.	n.d.	n.d.	p
5.	5.41	377.0422	359, 197, 161	Rosmarinic acid derivative	n.d.	n.d.	p	p	n.d.
6.	5.60	555.1807	537, 359, 295, 179	Salvianolic acid K derivative	p	p	n.d.	n.d.	p
7.	6.50	683.2615	537, 357, 197, 179, 161, 135	Salvianolic acid K methyl pentoside	n.d.	n.d.	n.d.	n.d.	p
8.	7.00	735.2613	537, 493, 359, 295	Lithospermic acid	p	p	p	p	p
9.	7.32	521.1120	359, 197, 179, 161, 135	Rosmarinic acid hexoside III	n.d.	n.d.	n.d.	n.d.	p
10.	7.80	719.2865	359, 197, 179, 161, 135	Sagerinic acid	n.d.	n.d.	p	p	n.d.
11.	8.01	717.2477	537, 519, 339, 321, 295, 197, 179	Salvianolic acid B	p	p	p	p	p
12.	8.30	359.0282	197, 179, 161, 135	Rosmarinic acid	p	p	p	p	p
13.	8.91	1074.6538	537, 359, 197, 179	Lithospermic acid dimer I	n.d.	n.d.	n.d.	n.d.	p
14.	9.38	1074.6538	537, 359, 197, 179	Lithospermic acid dimer II	n.d.	n.d.	p	p	n.d.
15.	9.72	569.1367	537, 359, 197, 179	Lithospermic acid derivative	p	p	n.d.	n.d.	n.d.
16.	9.81	493.0895	359, 197, 179	Salvianolic acid A	n.d.	n.d.	n.d.	n.d.	p
17.	10.37	717.2409	537, 359, 179	Salvianolic acid B isomer I	n.d.	n.d.	p	p	n.d.
18.	11.08	717.2438	519, 359, 179	Salvianolic acid B isomer II	p	p	n.d.	n.d.	n.d.
19.	11.79	313.1357	269, 203, 161, 135	Salvianolic acid F isomer I	p	n.d.	n.d.	n.d.	n.d.
20.	11.89	313.0336	161	Salvianolic acid F isomer II	n.d.	n.d.	p	p	n.d.

R1—roots from 1-year-old plants; R2—roots from 2-year-old plants; AP1—aerial parts from 1-year-old plants; AP2—aerial parts from 2-year-old plants; I—inflorescences from 2-year-old plants. p—compound present, identified, and quantified in the organ; n.d.—compound not detected.

**Table 2:** Content of phenolic compounds (mg/100 g D.W.) in methanolic-aqueous extracts from different organs (roots, aerial parts, and inflorescences) of *S*. *tomentosa* plants.

Peak No.	Tentative Identification	R1	R2	AP1	AP2	I
1.	Caffeic acid	0.27 ± 0.05 ^a^	0.17 ± 0.04 ^a^	0.62 ± 0.05 ^c^	0.41 ± 0.05 ^ac^	0.98 ± 0.08 ^b^
2.	Rosmarinic acid hexoside I	0.87 ± 0.003 ^a^	0.27 ±0.09 ^b^	1.12 ± 0.02 ^a^	4.63 ± 0.11 ^c^	0.00 ^b^
3.	Rosmarinic acid hexoside II	27.45 ± 0.12 ^a^	11.73 ± 1.11 ^b^	15.79 ± 1.11 ^d^	3.85 ± 0.3 ^e^	0.00 ^c^
4.	Salvianolic acid K derivative I	0.00 ^a^	0.00 ^a^	0.00 ^a^	0.00 ^a^	7.64 ± 0.26 ^b^
5.	Rosmarinic acid derivative	0.00 ^a^	0.00 ^a^	3.39 ± 0.22 ^a^	1.37 ± 0.05 ^b^	0.00 ^a^
6.	Salvianolic acid K derivative II	15.21 ± 0.1 ^a^	17.64 ± 0.13 ^b^	0.00 ^c^	0.00 ^c^	0.00 ^c^
7.	Salvianolic acid K methyl pentoside	0.00 ^b^	0.00 ^b^	0.00 ^b^	0.00 ^b^	15.63 ± 0.3 ^a^
8.	Lithospermic acid A	9.34 ± 0.13 ^a^	12.51 ± 0.66 ^a^	148.20 ± 5.60 ^c^	50.71 ± 0.64 ^d^	28.01 ± 0.38 ^b^
9.	Rosmarinic acid hexoside	0.00 ^b^	0.00 ^b^	0.00 ^b^	0.00 ^b^	20.21 ± 0.77 ^a^
10.	Sagerinic acid	0.00 ^a^	0.00 ^a^	4.04 ± 0.03 ^b^	2.03 ± 0.08 ^c^	0.00 ^a^
11.	Salvianolic acid B	181.90 ± 0.58 ^a^	121.10 ± 0.94 ^b^	226.00 ± 0.72 ^d^	346.51 ± 4.91 ^e^	82.62 ± 1.42 ^c^
12.	Rosmarinic acid	481.78 ± 0.8 ^a^	425.87 ± 2.83 ^b^	524.71 ± 1.31 ^c^	518.25 ± 4.43 ^c^	436.86 ± 1.71 ^ab^
13.	Lithospermic acid dimer I	0.00 ^a^	0.00 ^a^	0.00 ^a^	0.00 ^a^	153.57 ± 2.35 ^b^
14.	Lithospermic acid dimer II	0.00 ^a^	0.00 ^a^	201.76 ± 1.42 ^b^	1.38 ± 0.07 ^c^	0.00 ^a^
15.	Lithospermic acid derivative	0.91 ± 0.04 ^a^	3.45 ± 0.12 ^b^	0.00 ^c^	0.00 ^c^	0.00 ^c^
16.	Salvianolic acid A	0.00 ^b^	0.00 ^b^	0.00 ^b^	0.00 ^b^	34.63 ± 0.49 ^a^
17.	Salvianolic acid B isomer I	0.00 ^a^	0.00 ^a^	29.73 ± 2.5 ^b^	19.11 ± 2.92 ^c^	0.00 ^a^
18.	Salvianolic acid B isomer II	12.72 ± 0.15 ^a^	64.83 ± 1.84 ^b^	0.00 ^c^	0.00 ^c^	0.00 ^c^
19.	Salvianolic acid F isomer I	7.25 ± 0.5 ^a^	0.00 ^b^	0.00 ^b^	0.00 ^b^	0.00 ^b^
20.	Salvianolic acid F isomer II	0.00 ^a^	0.00 ^a^	42.76 ± 1.95 ^b^	15.30 ± 0.82 ^c^	0.00 ^a^
	Sum [mg/100 g D.W.]	737.70	657.57	1198.12	963.55	780.15

R1—roots of 1-year-old plants; R2—roots of 2-year-old plants; AP1—aerial parts from 1-year-old plants; AP2—aerial parts from 2-year-old plants; I—inflorescences from 2-year-old plants. The results are means from 3–4 samples ± standard error (SE). Means with the same letter within each line are not statistically different in the Kruskal–Wallis test at *p* ≤ 0.05.

**Table 3:** Antimicrobial activity of methanolic-aqueous extracts from aerial parts, roots, and inflorescences of *S*. *tomentosa*.

Strain	R1	R2	AP1	AP2	I	Rosmarinic Acid	Gentamicin	Fluconazole
Gram-positive bacteria	MIC/MBC (mg/mL)						MIC/MBC (µg/mL)	
*Staphylococcus aureus* ATCC 25923	2.5/2.5	2.5/2.5	5/5	5/5	5/5	1/>1	0.5	-
*Staphylococcus aureus* MM3 MRSA	2.5/2.5	2.5/2.5	2.5/2.5	2.5/2.5	2.5/2.5	>1/>1	2	-
*Staphylococcus aureus * ZMF MLSB	2.5/2.5	2.5/2.5	5/5	5/5	2.5/2.5	>1/>1	0.12	-
*Staphylococcus epidermidis* ATCC 12228	0.625/0.625	0.625/0.625	0.625/0.625	0.625/0.625	0.625/0.625	1/>1	16	-
*Staphylococcus haemolyticus * PCM 2113	2.5/2.5	2.5/2.5	>5/>5	>5/>5	2.5/2.5	1/>1	0.06	-
*Staphylococcus hominis* DSM 20328	5/5	5/5	>5/>5	>5/>5	2.5/2.5	1/>1	0.06	-
*Staphylococcus simulans * DSM 20723	>5/>5	>5/>5	>5/>5	>5/>5	>5/>5	1/>1	0.06	-
*Staphylococcus pseudintermedius * PCM 2791	2.5/2.5	2.5/2.5	5/5	5/5	5/5	1/>1	0.12	-
*Enterococcus feacalis* ATTC 29212	>5/>5	>5/>5	>5/>5	>5/>5	>5/>5	1/>1	16	-
*Enterococcus faecium * PCM 1859	>5/>5	>5/>5	>5/>5	>5/>5	>5/>5	1/>1	34	-
*Enterococcus hirae * ATCC 10541	>5/>5	>5/>5	>5/>5	>5/>5	>5/>5	1/>1	32	
*Bacillus cereus * PCM 1948	0.312/0.312	0.312/0.312	1.25/1.25	1.25/1.25	0.312/0.312	1/1	0.25	-
*Bacillus subtilis* ATCC 6635	2.5/2.5	2.5/2.5	5/5	5/5	2.5/2.5	1/1	0.25	-
*Listeria monocytogenes* PCM 2191	>5/>5	>5/>5	>5/>5	>5/>5	>5/>5	>1/>1	0.25	-
Gram-negative bacteria								
*Escherichia coli * ATCC 25922	2.5/2.5	2.5/2.5	2.5/2.5	2.5/2.5	2.5/2.5	1/1	2	-
*Escherichia coli * ZMF ESBL	>5/>5	>5/>5	>5/>5	>5/>5	>5/>5	>1/>1	128	-
*Pseudomonas aeruginosa* ATCC 27853	2.5/2.5	2.5/2.5	2.5/2.5	2.5/2.5	2.5/25.	1/>1	2	-
*Proteus vulgaris* CCM 1799	5/5	5/5	>5/>5	>5/>5	5/5	1/1	0.12	-
*Salmonella enteritidis* ZMF 279	>5/>5	>5/>5	>5/>5	>5/>5	>5/>5	>1/>1	0.25	-
*Shigella sonnei * PCM 2466 FII	5/5	5/5	5/5	5/5	5/5	>1/>1	5	-
Fungi								
*Candida albicans* ATTC 10231	2.5/2.5	2.5/2.5	2.5/2.5	2.5/2.5	2.5/2.5	1/>1	-	5/>5
*Aspergillus brasiliensis* ATCC 16404	>5/>5	>5/>5	>5/>5	>5/>5	>5/>5	1/>1	-	5/>5

R1—roots from 1-year-old plants; R2—roots from 2-year-old plants; AP1—aerial parts from 1-year-old plants; AP2—aerial parts from 2-year-old plants; I—inflorescences from 2-year-old plants; gentamicin—wide-spectrum antibacterial antibiotic: fluconazole—antifungal chemotherapeutic. MRSA—methicyllin-resistant *Staphylococcus aureus*; MLSB—resistance to macrolide; lincosamide and streptogramin B; ESBL—extended-spectrum beta-lactamases; MIC—minimal inhibitory concentration; MBC—minimal bactericidal concentration.

## Data Availability

Data are contained within this article.
